# Repeat Sentinel Lymph Node Biopsy for Ipsilateral Breast Tumor Recurrence After Breast Conserving Surgery With Sentinel Lymph Node Biopsy: Pooled Analysis Using Data From a Systematic Review and Two Institutions

**DOI:** 10.3389/fonc.2020.518568

**Published:** 2020-09-23

**Authors:** Chang Ik Yoon, Sung Gwe Ahn, Dooreh Kim, Jung Eun Choi, Soong June Bae, Chi Hwan Cha, Soeun Park, Joon Jeong

**Affiliations:** ^1^Division of Breast Surgery, Department of Surgery, College of Medicine, Seoul St Mary's Hospital, The Catholic University of Seoul, Seoul, South Korea; ^2^Department of Surgery, Gangnam Severance Hospital, Yonsei University College of Medicine, Seoul, South Korea; ^3^Department of Surgery, College of Medicine, Yeungnam University, Daegu, South Korea

**Keywords:** repeat sentinel lymph node biopsy, false negative rate (FNR), recurrent breast cancer, identification rate (IR), SLNB

## Abstract

**Introduction:** Best surgical approach of axillary staging remains controversial in locally recurrent breast cancer. We evaluated the reliability of repeat sentinel lymph node biopsy (reSLNB) in patients with ipsilateral breast tumor recurrence (IBTR) after breast conserving surgery (BCS) with sentinel lymph node biopsy (SLNB) in terms of identification rate (IR) and false negative rate (FNR). To address the FNR, we identified patients who underwent sequential axillary lymph node dissection (ALND) after reSLNB.

**Methods:** A systematic search of PubMed, EMBASE, and Cochrane Library were conducted to identify patient-level data from articles. We searched for data of patients who underwent BCS with SLNB for primary breast cancer and who underwent sequential ALND after reSLNB due to local recurrence. Patients data was also identified by the same criteria at two institutions.

**Results:** In total, 197 peer-reviewed publications were obtained, of which 20 included patients who met the eligibility criteria. Data from 464 patients were collected. From the two institutions, 31 patients were identified. A total of 495 patients were pooled. The IR of reSLNB was 71.9% (356/495). To address the FNR of reSLNB, 171 patients who underwent ALND after reSLNB were identified. The FNR and accuracy of reSLNB were 9.4% (5/53) and 97.1% (165/170), respectively.

**Conclusion:** Our pooled data analysis showed that the FNR of reSLNB is lower than 10%, indicating that this operation is a reliable axillary surgery in patients with IBTR after they underwent BCS.

## Introduction

Metastasis in axillary lymph nodes is the most important prognostic factor in patients with breast cancer (1). In the past, axillary lymph node dissection (ALND) has been the standard approach for axillary surgery in breast cancer. However, ALND is associated with short-term and long-term morbidities (2–5). Patients treated with sentinel lymph node biopsy (SLNB) have significantly lower post-operative complication such as lymphedema, infection, seroma, and numbness compared to those with ALND (6). Among these complication, lymphedema is one of the most common complication after ALND, and adversely affects the quality of life. Despite of different definition and measurement, the incidence of ALND has been reported up to 56% (7). Nevertheless, the benefits of ALND are limited because most patients with early stage breast cancer are node-negative. SLNB is a less invasive procedure; it can replace ALND in patients with clinically node-negative breast cancer. SLNB has been reported to have a >90% identification rate (IR) and <10% false-negative rate (FNR) (8, 9). Previous studies have reported that SLNB can accurately predict the status of the remaining axillary lymph nodes (10–12).

Because of these advantages, SLNB plays an integral role in the axillary staging for the surgical management of patients with early breast cancer. However, the role of SLNB remains controversial in the surgical management of patients with local recurrence. Ipsilateral breast tumor recurrence (IBTR) after breast conserving surgery (BCS) as the initial surgery has gradually increased; this happens because BCS is currently performed in two of every three cases of surgery for primary breast cancer (13). The 10 year-local recurrence rate after BCS or mastectomy has been reported to be 2–10% (14–16).

For removal of recurred breast lesions in remained breast after BCS, total mastectomy or second lumpectomy can be performed (17). For concurrent axillary surgery, repeat SLNB (reSLNB) might be considered (18, 19). However, most patients with IBTR have a history of undergoing SLNB and radiotherapy that could interrupt their lymphatic channels. Evidence concerning the role of reSLNB for IBTR is still lacking despite the results of previous studies (20–23). The vast majority of earlier studies included few patients and had a retrospective design. In addition, studies that included patients who underwent mastectomy or ALND were heterogeneous (21–23).

In the present study, we focused on the reliability of reSLNB in patients who underwent BCS and SLNB without ALND as the initial surgery. To address the FNR of reSLNB, we further identified patients with IBTR who underwent ALND after reSLNB. To achieve this goal, we conducted a pooled analysis using data from a systematic review and two institutions.

## Methods

### Search Strategy

A literature search was performed in PubMed, Embase, and Cochrane Library databases of systemic review. All articles including case reports and original articles were searched. These articles were found using the following search terms in the databases: “ipsilateral breast tumor recurrence,” “locally recurrent breast cancer,” “recurrent breast cancer,” “sentinel lymph node biopsy,” “lymphatic mapping,” “repeat,” and “re-operative” (see Search Terms). The articles were independently selected by two researchers, and the literature search was conducted until April 2018.

### Definition, Inclusion Criteria, and Data Extraction for reSLNB

IBTR was defined as recurred breast tumors or new ipsilateral primary breast tumors because it was impossible to distinguish the two diagnoses. A positive reSLNB outcome was defined as the presence of micro-metastasis (>0.2 mm and/or >200 cells, but not larger than 2 mm) and macro-metastasis, according to the American Joint Committee on Cancer, 7th edition (24). Isolated tumor cells (clusters of cells <0.2 mm and/or <200 cells) were defined as node-negative. The most selected articles did not specify exact radiation field and dose. Thus, we excluded analysis of radiotherapy because we could not distinguish whether a patient received radiation on the breast and/or axilla.

Patients included in this analysis had to meet the following criteria: (i) history of BCS for former breast cancer or ductal carcinoma *in situ* with histologically clear margins and of SLNB without ALND, (ii) IBTR or new ipsilateral primary breast tumor, and (iii) reSLNB and sequential ALND to assess the FNR of reSLNB. The following cases were excluded from the analysis: (i) presence of distant metastasis, and (ii) presence of inflammatory breast cancer. Even if the first and second operations were performed at different hospitals, patients were included if medical records from both hospitals were confirmed (see Information data extraction). With the corrected data, we attempted to answer the question (see Review questions).

**Figure 1:**
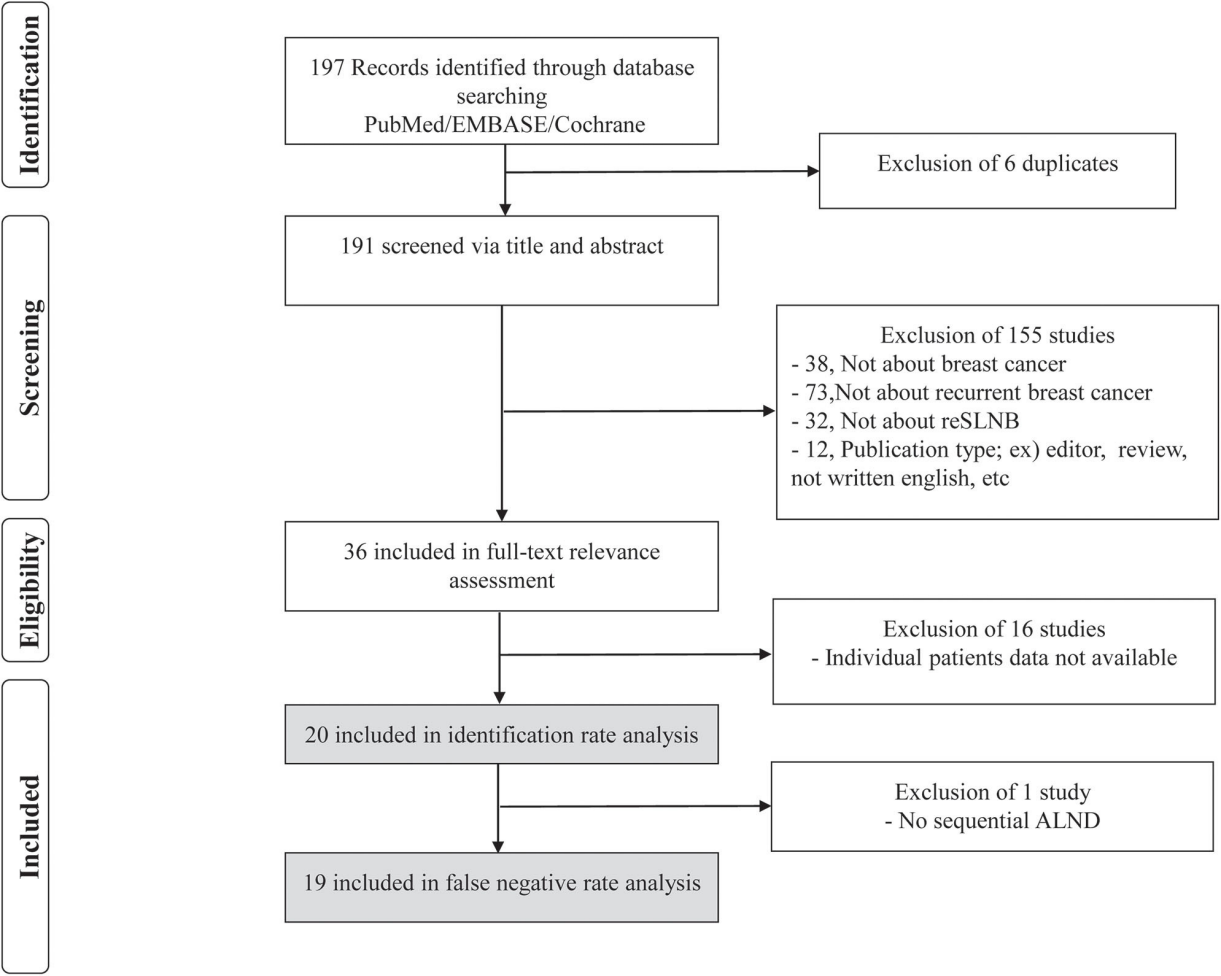
Flowchart for the search strategy on repeat sentinel lymph node biopsy in patients with local recurrence. Our search strategy found 197 abstracts. Of these 197 abstracts, 6 were excluded due to duplication, and 155 were excluded based on predefined exclusion criteria. The remaining 36 articles were fully reviewed, of which 16 were excluded because they failed to meeting inclusion criteria.

This study was guided by the Preferred Reporting Items for Systemic Reviews and Meta-analyses (PRISMA) statement (25). The selection process with PRISMA standards in our study is depicted in Figure 1. All articles were searched independently by Chang Ik Yoon and Sung Gwe Ahn. In the literature search, patient-level data were collected. Articles not published in English, articles in which full-text articles were unavailable, review articles, duplicated articles, commentaries, editorials, poster, conference papers, and letters to the editor were excluded. Discrepancies were resolved through discussion (Chang Ik Yoon and Sung Gwe Ahn). Data obtained from the literature search and two institutions, Gangnam Severance Hospital and Yeungnam University Hospital, were analyzed together. Inclusion and exclusion criteria for patient data from the two institutions were the same as those mentioned above. The injection methods, doses, and sites for lymphatic mapping varied among studies (Table 1 and Supplementary Table 1). The injection and SLNB protocol at Gangnam Severance Hospital and Yeungnam University was as follows. A radioisotope was injected into the subdermal layer of the periareolar site 15 min before surgery. Sentinel lymph nodes (SLNs) were identified and removed using a Gamma-ray detecting probe. Sequential ALND was performed in all patients after reSLNB. Breast surgery was performed with second lumpectomy or mastectomy. Harvested SLNs were fixed using 10% formalin solution. Each SLN was sectioned into 2–3-mm-thick slices, and all slices were frozen and examined microscopically.

**Table 1:** Information of publication year, number of patients, identification rate and pathologic status for 20 studies on repeat sentinel lymph node biopsy (reSLNB).

**References**	**Years**	**No**.	**Identification rate**	**TP**	**TN**	**FN**	**Injection site of radioisotope**
^*^Vugts et al. (23)	2015	179	60.3% (108/179)	9	29	2	Intratumoral, periareolar, peritumoral
^#^Intra et al. (18)	2014	36	100% (36/36)	6	0	0	Intraparenchymal, subareolar, subdermal
^#^Dinan et al. (26)	2005	2	100% (2/2)	0	1	0	Intradermal, peritumoral, subareolar
^#^^,^^†^^,^^^*^^Boughey et al. (27)	2006	5	100% (5/5)	1	4	0	Intratumoral, peritumoral
^*^Jackson et al. (28)	2006	1	100% (1/1)	1	0	0	Subareolar
^*^Newman et al. (29)	2007	2	50% (1/2)	0	1	0	Subareolar
^*^Roumen et al. (30)	2006	2	100% (2/2)	1	0	0	Intratumoral, peritumoral
^*^Taback et al. (31)	2006	6	83.3% (5/6)	0	5	0	Intratumoral
^*^Port et al. (32)	2007	54	74.1% (40/54)	5	22	1	Intradermal
^*^Cox et al. (33)	2008	55	81.8% (45/55)	9	0	0	Intraparenchymal, subareolar
^#^Koizumi et al. (34)	2007	3	66.7% (2/3)	1	1	0	Intraparenchymal, intradermal
^*^Schrenk et al. (35)	2007	11	90.9% (10/11)	0	10	0	Intraparenchymal
^*^Tasevki et al. (36)	2009	1	100% (1/1)	0	1	0	Intraparenchymal, subareolar, subdermal
^§^Derkx et al. (37)	2010	12	33.3% (4/12)	0	1	0	NR
^*^Tokmak et al. (38)	2014	5	60% (3/5)	0	3	0	Intradermal, periareolar, peritumoral
^#^Cordoba et al. (39)	2014	10	50% (5/10)	1	4	0	Subareolar
^*^Matsuomoto et al. (40)	2015	22	81.8% (18/22)	1	0	0	Intradermal, peritumoral
^*^Karanlik et al. (41)	2016	21	81.0% (17/21)	6	0	0	Intradermal, peritumoral
^#^Folli et al. (42)	2016	30	76.7% (23/30)	2	20	1	Peritumoral, subdermal
^#^,^*^Barone et al. (43)	2007	7	100% (7/7)	NR	NR	NR	Intraparenchymal, subareolar
Total		464	72.2% (335/464)	43	102	4	
^#^Two institutions^a^		31	67.7% (21/31)	5	15	1	Subareolar, intradermal
Total of pooled-analysis		495	71.9% (356/495)	48	117	5	

*FN, false negative; IBTR, ipsilateral breast tumor recurrence; No., Number; NR, not recorded; TN, true negative; TP, true positive; SLNB, sentinel lymph node biopsy*.

a *Two institutions: Gangnam Severance Hospital and Yeungnam University*.

Mapping method of SLNB:

# *radioisotope only*,

† *blue dye only*,

* *combined blue dye and radioisotope*,

§ *unknown*.

The study was conducted in accordance with the good clinical practice guidelines and the Declaration of Helsinki, and the protocol was approved by the Institutional Review Board of Gangnam Severance Hospital (Local IRB number: 3-2018-0344).

### Statistical Analysis

IR of reSLNB was defined as the number of successful cases divided by the total number of patients who underwent reSLNB. The FNR, accuracy, true-positive rate, and negative predictive value of reSLNB were calculated, respectively. The FNR of reSLNB is considered too high if the FNR is <10%. Differences in IR according to the mapping methods (dual mapping/radioisotope only/blue dye only) were compared using the chi-square test. SPSS version 23 (SPSS, Inc., Chicago, IL, USA) was used for statistical analyses. Statistical significance was defined as *p*-value of <0.05.

## Results

### Search Results

In PubMed, EMBASE, and Cochrane database, we found 194 articles using the above mentioned searching terms (Figure 1). All articles retrieved from Embase and Cochrane Library databases were included to those extracted from PubMed. Adding the three articles from references in the previous meta-analysis of reSLNB (21, 22), a total of 197 articles were initially identified. Of these, there were six duplicated articles. A total of 191 abstracts were reviewed, and 155 abstracts were excluded for the following reasons: (i) not about breast cancer (*n* = 38), (ii) not about recurrent breast cancer (*n* = 73), (iii) not about reSLNB (*n* = 32), (iv) inappropriate publication types such as editorial, review, or articles not written in English (*n* = 12). A full-text review of 36 articles was conducted. A total of 20 articles finally met the inclusion criteria. From these, 19 articles analyzed the FNR of reSLNB. These articles were published from 2005 to 2016 (Table 1).

In addition, from 1995 to 2017, a total of 31 patients with IBTR after BCS met the inclusion criteria in the Gangnam Severance Hospital and Yeungnam University Hospital databases. These patients underwent reSLNB for their axillary staging.

### Identification Rate of reSLNB

**Figure 2:**
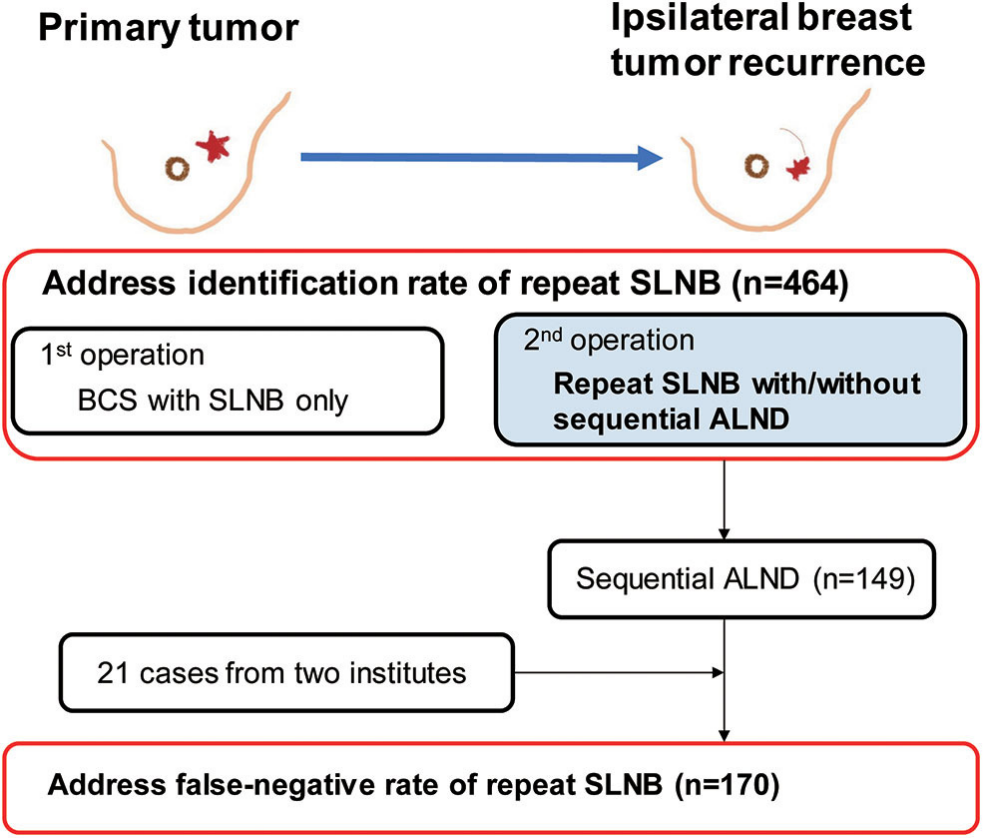
Flowchart for data curation in order to address the identification rate and false-negative rate of repeat sentinel lymph node biopsy. A total of 1,212 cases of 20 articles were found, 464 subjects met inclusion criteria about first breast cancer surgery. Among these, repeat sentinel lymph node was performed in 335 patients, resulting in 72.2% of identification rate for reSLNB. Except 1 article, sequential ALND was performed in 149 patients in 19 articles. Then we added 21 patients who underwent sequential ALND after reSLNB from two institutions. Finally, a total of 170 patients were analyzed for false-negative rate of reSLNB. BCS, breast conserving surgery; SLNB, sentinel lymph node biopsy; ALND, axillary lymph node dissection.

A total of 464 cases of reSLNB were found in the literature search (Figure 2). Of these, 335 were successful. The IR of reSLNB in articles was 72.2% (335/464). Among the 31 cases of reSLNB performed at the two institutions, 10 cases of sentinel failure occurred. The IR was 67.7% (21/31). The total IR of the pooled analysis was 71.9% (356/495) (Table 1).

The IR according to mapping tracers was described in Supplementary Table 2. The IR of dual mapping was 69.9% (251/359) and that of single mapping with a radioisotope was 79.5% (89/112). In three other reports where tracers were not clearly distinguished, the IR was 66.7%. There was no significant difference in IR according to mapping tracers (Supplementary Table 2, *p* = 0.122).

### FNR/Diagnostic Performance of reSLNB

In 19 articles, the results of ALND following reSLNB were obtained from patient-level data of 149 patients (Figure 2). True positive-, false negative-, and true negative cases of reSLNB were 43, 4, and 102, respectively (Table 1). In addition, data from 21 patients with sequential ALND of the two institutions were added.

In a total of 170 patients who underwent ALND after reSLNB, the overall FNR was 9.4% (5/53) (Table 2). The overall accuracy, true positive rate (same as sensitivity), and negative predictive value of reSLNB were 97.1, 90.6, and 95.9%, respectively (Table 2). The FNRs of reSLNB using single or dual tracers were 11.8% (2/17) and 8.6% (3/35), respectively (Table 3). There were no statistically significant differences in FNR of reSLNB according to mapping method (Table 3, *p* = 0.886).

**Table 2:** Pathologic status of sentinel and axillary lymph nodes in the patients with reSLNB.

		**Axillary lymph nodes**	
		**Positive**	**Negative**
SLNs^†^	Positive	48 (TP)	0
	Negative	5 (FN)	117 (TN)

*FN, false negative; TN, true negative; TP, true positive; SLNs, sentinel lymph nodes*.

† *False-negative rate: FN/(TP+FN) = 5/(48 + 5) = 9.4%; Sensitivity: TP/(TP+FN) = 48/48 + 5 = 90.6%; negative predictive value: TN/(TN+FN) = 117/117+5 = 95.9%; overall accuracy: (TP+TN)/number of patients = (48 + 117)/170 = 91.7%*.

**Table 3:** False negative rate (FNR) of reSLNB according to mapping methods.

**Mapping methods of reSLNB**	**No. of study (*n* = 19)**	**No. of cases (*n* = 170^**#**^)**	**FNR**	***p*-value**
				0.886
Dual mapping methods	12	106	8.6% (3/35)	
Radioisotope only	5	58^**†**^	11.8%^**†**^ (2/17)	
Blue dye only	0	0		
Not clearly distinguished^§^	2	6	0% (0/1)	

*IR, identification rate; No, number; SLNB, sentinel lymph node biopsy*.

† *combined data of articles and two institution*.

§ *the mapping methods of repeat-SLNB were not described or applied differently in each case, not included in statistical analysis*.

## Discussion

In primary breast cancer with early stage, SLNB has been preferred procedure for axillary staging because it offers oncologic safety with fewer complications such as lymphedema, pain, range of motion, and sensory defect compared to ALND (5, 44, 45). However, in locally recurrent breast cancer, it lacks evidence that reSLNB could be performed for axillary staging method in terms of FNR. Since a large multi-institutional randomized study showed that the FNR of SLNB was 9.8% in clinically node-negative breast cancer (4), recent trials aimed to demonstrate a safety of SLNB if the FNR wound not be >10% in clinically node-positive breast cancer treated with preoperative chemotherapy (46–48). On the basis of these studies, we consider that reSLNB would be acceptable if the FNR of reSLNB is not >10%.

With this background, our pooled analysis used abundant data concerning reSLNB performed in patients with IBTR and demonstrated that the procedure was reliable. The FNR was 9.4%, and followed by an accuracy of 97.1% and a negative predictive value of 95.9%, although the IR was low as 71.9%

Our study has several strengths compared with previous studies evaluating reSLNB for locally recurred breasts cancer. The FNR was accurately addressed as back-up ALND was conducted after successful reSLNB in about half of patients. To address the FNR of SLNB, ALND is an inevitable procedure to rule out the chance of metastases in lymph nodes not retrieved by SLNB. However, the vast majority of patients included in earlier studies underwent axillary staging through reSLNB alone. An accurate assessment of FNR of reSLNB by sequential ALND for patients with IBTR is the novelty of our study.

In addition, we only included patients with true IBTR. Our study population underwent BCS and SLNB alone for their primary breast cancer. The homogeneity of the first surgery is distinguished from that in other studies including patients who underwent mastectomy or ALND. Moreover, we enrolled a relatively large number of patients from previously published articles and local institutional database that has strength in delicate information of patients.

Traditionally, in patients with IBTR, complete axillary clearance has been considered essential, regardless of axillary nodal involvement. However, recent advances in non-invasive diagnostic imaging have raised questions against whether sequential ALND is mandatory because more than half of the patients with IBTR had no axillary metastases (21, 22). Thus, many investigators have interests in de-escalating axillary surgery, and may accept the concept of limited axillary management (19), as long as credible sentinel lymph node detection is guaranteed. Because, in cases of IBTR, preceding axillary surgery may lead to disruption of lymphatic flow that undermines reliability of reSLNB. Also, another study reported that aberrant lymphatic drainage was visualized in two-fifths of the patients with locally recurred cancer (43.2%) (21).

However, SLNB is a minimally invasive method and has a lower chance of fully destroying common lymphatic routes from the breast to axillary SLNs than ALND. It is at least indirectly supported by the results of a previous study in which the rate of aberrant lymphatic drainage was significantly lower in patients with a history of SLNB than in those with a history of ALND (17.4 vs. 69.2%) (21).

Moreover, some studies provided evidence that a few common afferent lymphatic channels exist and drain breast tumors to axillary SLNs through several major lymphatic trunks (49, 50), implying a possibility of an alternative path from the breast to axillary lymph nodes after previous axillary surgery. Our data showed that reSLNB was successfully performed in 71.9% of the patients with IBTR, suggesting that lymphatic tracts between the breast and SLNs are intact in more than two-third of patients undergoing previous SLNB. As a consequence, reSLNB could be more reliably performed in patients with a history of SLNB alone.

A fundamental limitation of this study was the heterogeneity among the included studies. There are several differences such as surgical techniques, mapping methods of SLNB, and radiation therapy. Regarding prior radiotherapy affecting lymphatic drainage, information was missed in most patients from the articles, although a majority of patients might be treated with radiotherapy after breast conservative surgery. In addition, most articles had very few patients and a retrospective design. Also, we did not perform a statistical analysis to confirm heterogeneity among studies due to the study design of pooled data analysis which collected data of identifiable patients in each study. Despite these limitations, our study was a large-scale pooled analysis that showed that reSLNB is reliable for axillary staging in patients with IBTR and who were formerly treated with BCS and SLNB.

In conclusion, our study found that the reSLNB FNR is lower than 10% indicating that this procedure is reliable for axillary staging in patients with IBTR, even though they already underwent SLNB. It could be a feasible axillary surgery in these patients like those with primary cancer. Further validation through prospectively designed studies is warranted for these findings.

## Data Availability Statement

All datasets generated for this study are included in the article/Supplementary Material.

## Ethics Statement

The study was conducted in accordance with the good clinical practice guidelines and the Declaration of Helsinki, and the protocol was approved by the Institutional Review Board of Gangnam Severance Hospital (Local IRB number: 3-2018-0344). The need for informed consent was waived under the approval of the IRB due to the retrospective design.

## Author Contributions

CY, SA, and JJ contributed conception and design of the study. CY, SA, JC, and SB organized the database. DK, CC, and SP assisted to first draft of the manuscript. All authors contributed to manuscript revision, read, and approved the submitted version.

## Search Terms in the Databases

### In PubMed

((((((ipsilateral breast tumor recurrence) OR locally recurrent breast cancer) OR recurrent breast cancer)) AND (((“Sentinel Lymph Node Biopsy”) OR sentinel lymph node biopsy) OR lymphatic mapping))) OR (((((“Sentinel Lymph Node Biopsy”) OR sentinel lymph node biopsy) OR lymphatic mapping)) AND ((repeat) OR re-operative)).

### In Embase

(ipsilateral AND breast AND tumor AND recurrence OR (locally AND recurrent AND breast AND cancer) OR (recurrent AND breast AND cancer)) AND (sentinel AND lymph AND node AND biopsy OR (lymphatic AND mapping)) AND (repeat OR “re operative”).

### In Cochrane Library Database

((ipsilateral breast tumor recurrence) OR (locally recurrent breast cancer)) OR ((recurrent breast cancer) OR (sentinel lymph node biopsy) OR (lymphatic mapping)) AND ((repeat) OR (re-operative)).

### Information Data Extraction: The Following Information Was Collected

1. Number of patients with ipsilateral breast tumor recurrence (locally recurrent breast cancer)
2. Primary breast treatment: mastectomy with breast conserving surgery/lumpectomy
3. Primary axillary treatment: sentinel lymph node biopsy (SLNB), axillary lymph node dissection (ALND), or none
4. Adjuvant radiotherapy after primary event
5. Secondary axillary treatment: repeat sentinel lymph node biopsy (reSLNB), sequential ALND, or none
6. Mapping methods of reSLNB
7. Identification rate and false negative rate of reSLNB
8. Pathologic status of reSLNB
9. Sequential ALND and pathologic outcome.

### Review Questions: With the Extracted Data, an Attempt Was Made to Answer the Following Questions

1. What is the identification rate for a reSLNB?
2. What is the mapping methods for the reSLNB procedure?
3. What is pathologic status of the repeat sentinel lymph node (reSLN)?
4. What is the false-negative rate and the identification of reSLNB?

## Conflict of Interest

The authors declare that the research was conducted in the absence of any commercial or financial relationships that could be construed as a potential conflict of interest.

## Abbreviations

reSLNB: repeat sentinel lymph node biopsy
IBTR: ipsilateral breast tumor recurrence
BCS: breast conserving surgery
SLNB: sentinel lymph node biopsy
IR: identification rate
FNR: false negative rate
ALND: axillary lymph node dissection
PRISMA: Preferred Reporting Items for Systemic Reviews and Meta-analysis
SLN: sentinel lymph node.
