# Supplementary material for: Repeat Sentinel Lymph Node Biopsy for Ipsilateral Breast Tumor Recurrence After Breast Conserving Surgery With Sentinel Lymph Node Biopsy: Pooled Analysis Using Data From a Systematic Review and Two Institutions
Source: Front Oncol. 2020 Sep 23;10:518568. doi: 10.3389/fonc.2020.518568 (PMC7538804; doi:10.3389/fonc.2020.518568)
Supplement: Supplementary file 1 [file Data_Sheet_1.docx]

**Supplementary Table 1. The injection doses on patients with reSLNB**

| **Study** | **Years** | **^*^Radiotracer (mCi)** |
| --- | --- | --- |
| **Vugts G et al.(23)** | 2015 | NR |
| **Intra M et al.(18)** | 2014 | 0.54 |
| **Dinan D et al.(28)** | 2005 | 0.11-1.22 |
| **Boughey JC et al.(29)** | 2006 | 0.5-2.5 |
| **Jackson BM et al.(30)** | 2006 | 1 |
| **Newman LA et al.(31)** | 2007 | 4 |
| **Roumen RM et al.(32)** | 2006 | 0.5-2.5 |
| **Taback B et al. (33)** | 2006 | 0.5 |
| **Port ER et al.(34)** | 2007 | 0.1-0.5 |
| **Cox CE et al.(35)** | 2008 | NR |
| **Koizumi M et al.(36)** | 2007 | 1.62 |
| **Schrenk P et al.(37)** | 2007 | 0.27-1.08 |
| **Tasevki R et al.(38)** | 2009 | NR |
| **Derkx F et al.(39)** | 2010 | NR |
| **Tokmak H et al.(40)** | 2014 | 0.8-1 |
| **Cordoba O et al.(41)** | 2014 | NR |
| **Matsuomoto A et al.(42)** | 2015 | NR |
| **Karanlik H et al.(43)** | 2016 | 1 |
| **Folli S et al.(44)** | 2016 | 0.54 |
| **Barone JL et al.(45)** | 2007 | NR |
| **Gangnam Severance** |  | 0.5 |
| **Yeungnam University** |  | 1 |

NR, not recorded; ^*^MBq: calculated by converting 1mCi=37MBq

**Supplementary Table 2. Identification rates (IR) of reSLNB according to mapping methods**

| **Mapping methods of reSLNB** | **No. of study (n=20)** | **No. of cases (n=495)** | **IR** | ***p* value** |
| --- | --- | --- | --- | --- |
|  |  |  |  | 0.122 |
| **Dual mapping methods** | 12 | 359 | 69.9% (251/359) |  |
| **Radioisotope only** | 5 | 112**^†^** | 79.5%**^†^** (89/112) |  |
| **Blue dye only** | 0 | 0 |  |  |
| **Not clearly distinguished^§^** | 3 | 24 | 66.7% (16/24) |  |

IR, identification rate; No, number; SLNB, sentinel lymph node biopsy

**^†^**combined data of articles and two institution

^§^the mapping methods of repeat-SLNB were not described or applied differently in each case, not included in statistical analysis
